# Functional Hydrogels with Chondroitin Sulfate Release Properties Regulate the Angiogenesis Behaviors of Endothelial Cells

**DOI:** 10.3390/gels8050261

**Published:** 2022-04-21

**Authors:** Haonan Wang, Qian Li, Yongchao Jiang, Xiaofeng Wang

**Affiliations:** 1 School of Mechanics and Safety Engineering, Zhengzhou University, Zhengzhou 450001, China; whn18838970880@163.com (H.W.); xiaofengwang@zzu.edu.cn (X.W.); 2 School of Materials Science and Engineering, Zhengzhou University, Zhengzhou 450001, China

**Keywords:** hydrogel, chondroitin sulfate, endothelial cell, angiogenesis

## Abstract

Functional hydrogels with properties that mimic the structure of extracellular matrix (ECM) and regulate cell behaviors have drawn much attention in biomedical applications. Herein, gelatin-based hydrogels were designed and loaded with chondroitin sulfate (CS) to endow biological regulation on the angiogenesis behaviors of endothelial cells (ECs). Manufactured hydrogels containing various amounts of CS were characterized via methods including mechanical tests, cytocompatibility, hemolysis, and angiogenesis assays. The results showed that the prepared hydrogels exhibited excellent mechanical stability, cytocompatibility, and hemocompatibility. Additionally, the angiogenesis behaviors of ECs were obviously promoted. However, excessive loading of CS would weaken the effect due to a higher proportion of occupation on the cell membrane. In conclusion, this investigation highlights the great potential of these hydrogels in treating ischemic diseases and accelerating tissue regeneration in terms of regulating the angiogenesis process via CS release.

## 1. Introduction

The vasculature is a key component of the tissue microenvironment due to its critical role in providing nutrients, oxygen, and signaling cues that influence biological outcomes [1,2]. Nowadays, ischemic diseases like atherosclerosis and diabetic foot have become a major cause of disability worldwide [3], because these diseases impede the normal behaviors of cells such as proliferation, migration, and interactions with pericytes [4,5]. A quick reconstruction of the vasculature is therefore needed to guarantee adequate exchange of nutrients and accelerated recovery of the abovementioned diseases [6].

Various attempts to date have focused primarily on exogenous growth factors or genetic modifications of cells to induce the angiogenesis process within tissue-engineered scaffolds [7]. However, recombinant angiogenic growth factors suffer from easy degradation and abnormal angiogenesis [8], while the gene-modified cells are difficult to implement due to uncontrollable behaviors. Thus, strategies to stimulate angiogenesis via functionalized scaffolds have been widely investigated [9,10].

It has been widely recognized that hydrogels with traits of a three-dimensional soft network and easy processibility are preferred in angiogenesis stimulation [11,12]. In a previous report by Choi et al., hydrogel scaffolds with small pores (<200 μm) favored the formation of vascular networks at high density and poor penetration depth, while those with large pores (>200 μm) presented the opposite trends [13]. In another study, a Cu-HHA/PVA@MΦ2 hydrogel scaffold was prepared by Liu et al. for synergistic improvement of impaired angiogenesis to accelerate the diabetic chronic wound healing [14]. These results indicate that the inherent properties and functionalization of hydrogel can be employed to induce the angiogenesis process.

As a complicated macromolecular sulfated polysaccharide from natural resources, as well as the major component of ECM [15], chondroitin sulfate (CS) has been approved to play multiple roles in regulating numerous activities of endothelial cells (ECs) [16,17]. For instance, a CS-coating method was employed to drive the endothelization of pericardium by Lopez-Moya et al. [18]. However, the effect of different CS concentrations was not further investigated. On the contrary, Xiong et al. fabricated a negatively charged alginate and chondroitin sulfate microsphere hydrogel (nCACSMH) with different CS loading [19]. Even though the authors indicated that nCACSMH can dramatically accelerate the angiogenesis-related genes in ECs, the angiogenesis behavior of ECs, i.e., the sprouting and formation of tubular structures, was not visibly detected, which might be attributed to the short release time of CS.

**Figure 1:**
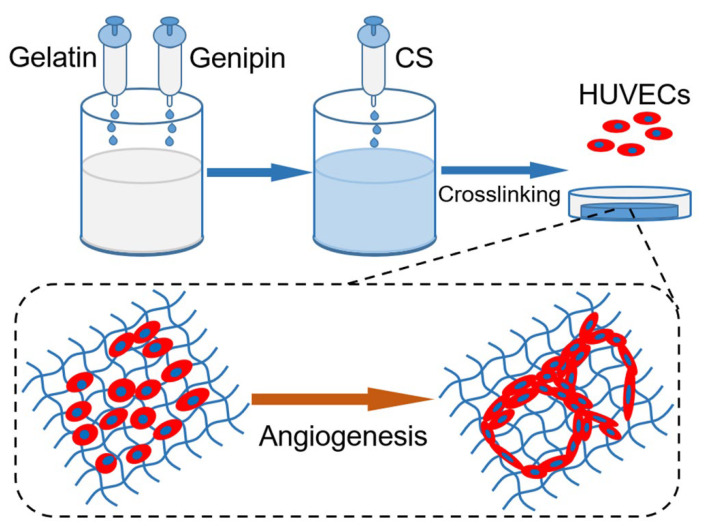
Schematic diagram of functional hydrogel fabrication.

Inspired by their conclusions, gelatin-based hydrogels with CS release properties are also proposed here, and are assumed to regulate the angiogenesis behaviors of ECs via various CS loading amounts. Different from the report by Xiong et al. [19], herein, gelatin hydrogel was chemically cross-linked by genipin during preparation, with the aim of maintaining its mechanical stability, prolonging the release period of CS, and the effective stimulation of ECs. Accordingly, the objective of this work was to develop a hydrogel platform with visible modulation on the angiogenesis behaviors of ECs (shown in Figure 1). Therefore, composite hydrogels containing various CS concentrations were manufactured and characterized on physical and biological tests.

## 2. Results and Discussion

### 2.1. Characterization of Hydrogels

**Figure 2:**
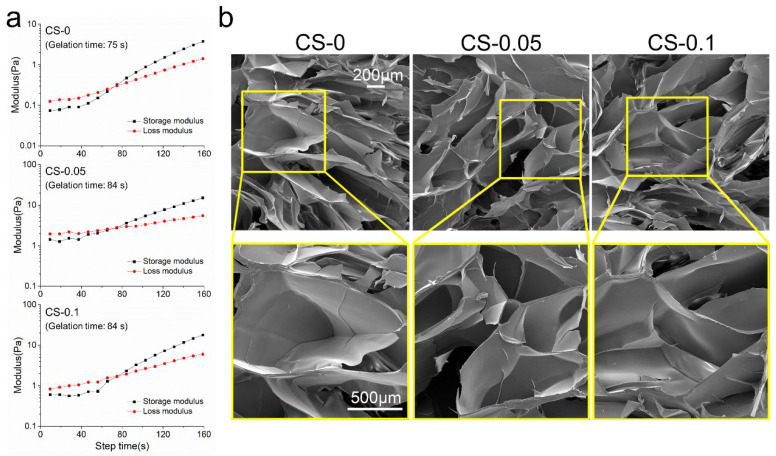
(**a**) The rheological behaviors, and (**b**) SEM images of different hydrogels.

The gelation behavior of different hydrogels was investigated, and the results are shown in Figure 2a. After 75 s at room temperature, gelatin began to gel due to the construction of hydrogen bonds within the molecular chains. Meanwhile, the gelation time increased to 84 s after CS addition, which might be ascribed to the charge repulsion between gelatin and CS molecules.

A quick reconstruction of the vasculature is necessary for adequate nutrient supply and metabolite exhaustion in biomedical fields, especially for engineered tissues [2]. To achieve this goal, the rapid and effective control of the angiogenesis process needs to be fully considered. It has been demonstrated that scaffolds with porous structures would benefit this process. From Figure 2b, it can be seen that all hydrogels show similar porous structures to the ECM. These kinds of hydrogels would be conducive to cell migration and penetration. Due to the relatively low amount of CS, there was no obvious change in structure among the groups. However, samples in the CS-0.05 and CS-0.1 groups shrank to some degree after CS addition. This can be attributed to the Hofmeister effect between gelatin and sulfate ions, which can remove the hydration water of the protein macromolecules from the hydrogels, leading to structure folding [20]. However, the decreased pore size accelerates the formation of vascular networks according to the results of Choi et al [13].

**Figure 3:**
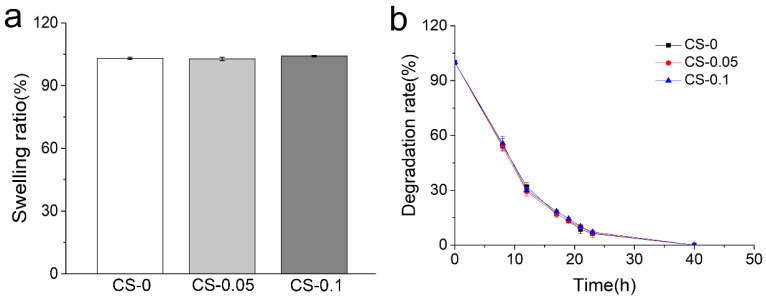
(**a**) Swelling ratios and (**b**) degradation rates of different hydrogels.

In general, gelatin-based hydrogels show high swelling properties and inferior resistance to body fluids. In a previous study by Entekhabi et al., gelatin hydrogel was reported to have a swelling ratio of 500% at the same concentration as our study [21]. Thus, a genipin crosslinker was employed here. In Figure 3a, hydrogels present swelling ratios of 103.10 ± 0.54%, 102.83 ± 0.76%, and 104.13 ± 0.35% in the CS-0, CS-0.05 and CS-0.1 groups, respectively, indicating the development of stability after crosslinking. Due to the collagenase enzymolysis, hydrogels of three groups can biodegrade within 40 h, as can be seen from Figure 3b. However, no obvious distinction can be found among the groups, meaning that the Hofmeister effect did not affect the swelling ratios and biodegradability of the hydrogels.

### 2.2. Loading and Release of CS

**Figure 4:**
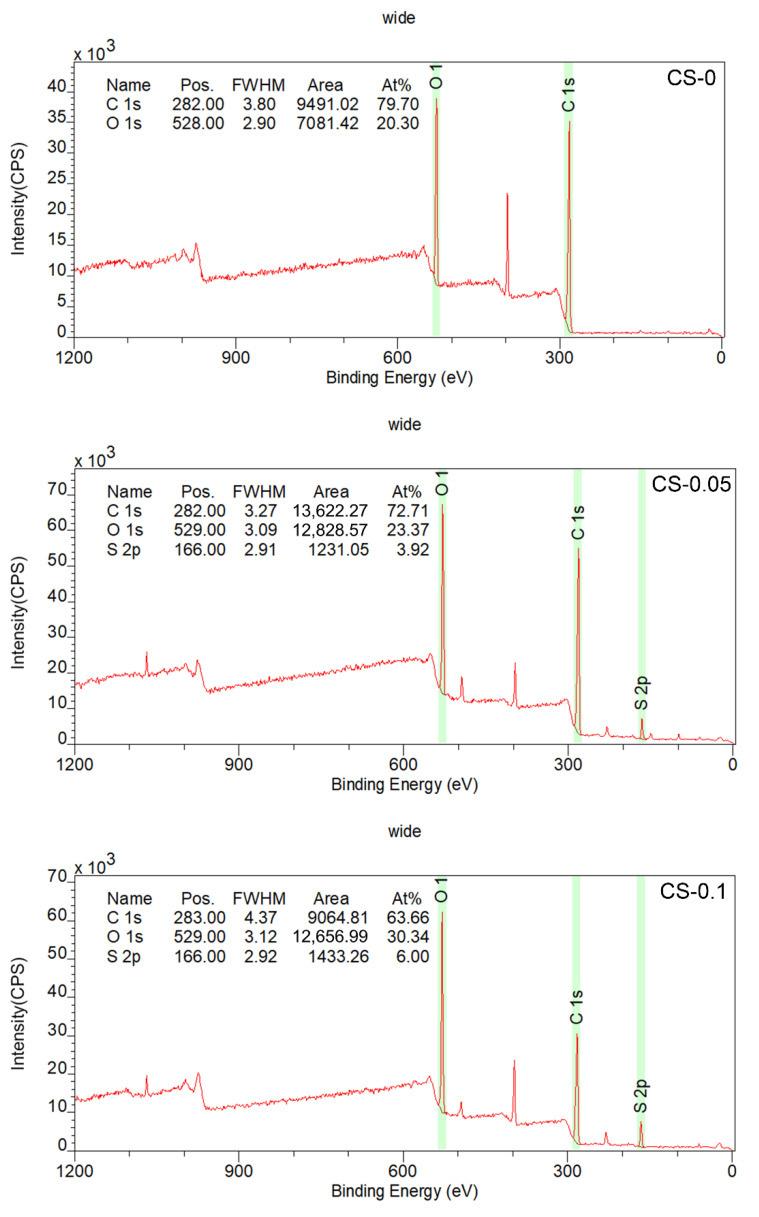
XPS spectra of the hydrogels.

The elemental spectra of the CS-0, CS-0.05, and CS-0.1 hydrogels were identified by XPS, and the results are displayed in Figure 4. As expected, S 2p photoelectron signals were only detected in the CS-0.05 and CS-0.1 groups. The atomic compositions of these samples were calculated on the basis of the XPS spectra, and are shown as inserts in Figure 4. According to the data, the elemental ratio of sulfur was 3.92 at% in the CS-0.05 sample, while it was 6.00 at% in the CS-0.1 group, nearly two-fold the value of the CS-0.05 sample. These results prove that CS was successfully introduced into the composite hydrogels.

**Figure 5:**
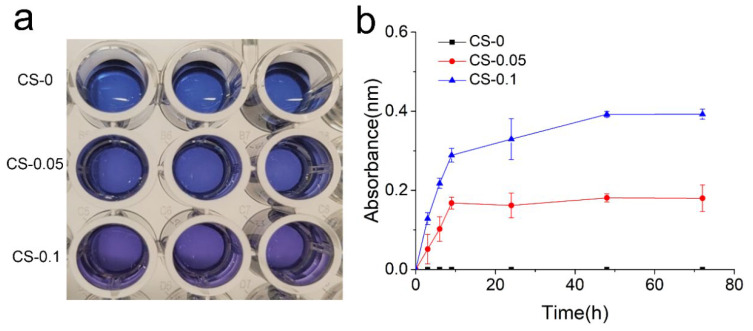
(**a**) Photographs of reactant, and (**b**) release profiles of CS in each group.

The release properties of CS from the hydrogels was quantified using the Azure A staining method. Azure A is a cationic dye, and a discoloration effect can occur through electrostatic interaction between Azure A and sulfate radicals in CS [22]. Therefore, the hydrogel extract, which is the initial blue color in the CS-0 group, changes to a bluish-purple color when Azure A is added to the CS-0.05 and CS-1 groups (Figure 5a). The release profiles of CS were determined, and are in Figure 5b. The profiles of the CS-0.05 and CS-1 groups are stable and linear during the first 10 h. The CS-0.1 group shows a higher release rate than CS-0.05, which ascribes to more loading amount of CS. The CS-0.05 hydrogel reaches an equilibrium state after 10 h. While in CS-0.1 group, the equilibrium state occurred after 48 h. The distinction may be attributed to electrostatic interactions between gelatin and CS. The data above also approve that the release behavior can be tuned through different CS loading.

### 2.3. Mechanical Properties of Hydrogels

**Figure 6:**
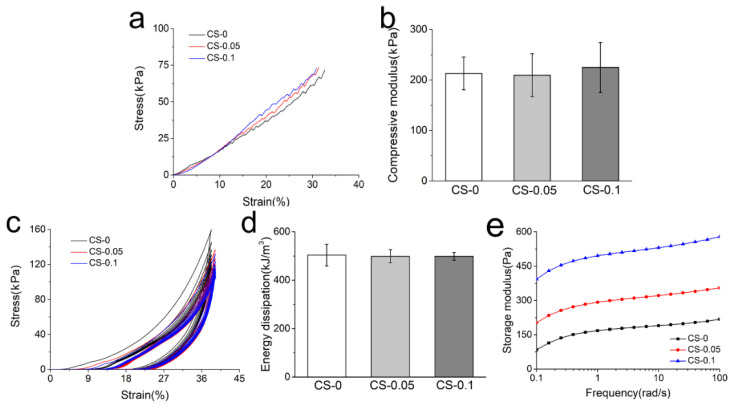
(**a**) The representative compressive stress–strain curves; (**b**) compressive modulus; (**c**) cyclic compressive stress–strain curves; (**d**) energy dissipation during cyclic compression on different hydrogels; and (**e**) storage modulus of each group.

The mechanical properties of hydrogels were studied using a universal testing machine, and the compressive stress–strain curves and corresponding moduli of different hydrogels are presented in Figure 6a,b. As can be observed, the compressive moduli of hydrogels are around 210 kPa, which is obviously higher than that presented by the pure gelatin hydrogel in our last report [23], indicating that the combination of genipin and CS can improve the mechanical properties of gelatin hydrogels. However, with 0.1% CS addition, the compressive modulus of CS-0.1 hydrogel only increased by tens of kPa compared to that of CS-0, meaning that the crosslinking effect is the main cause of the enhanced mechanical properties.

Cyclic compression and rheological testing were also carried out to study the mechanical properties of the hydrogels. The loading–unloading curves of composite hydrogels are presented in Figure 6c. All hydrogels are recoverable when the external force is removed, which is also attributed to the reinforcing effect of genipin and CS. Among these hydrogels, the dissipation energy (the area enclosed by loading and reloading curves) decreases slightly with CS addition in Figure 6d. Due to the dehydration and folding of gelatin macromolecules caused by the Hofmeister effect, the hydrogels adsorb less energy. However, the Hofmeister effect is not strong enough to cause an obvious decrease in energy dissipation. According to the rheological curves in Figure 6e, the storage modulus increased from around 180 kPa to around 530 kPa with CS addition. It is noteworthy that these storage moduli were much higher than those in Figure 2a. In the beginning, the hydrogen bonds mainly contributed to hydrogel formation, which was weak but quick. After 5 min of reaction, the genipin crosslinking was dominant, and the degree of crosslinking increased with time increment. When the crosslinking reactions were completed after 24 h, no significant difference in mechanical properties could be found, as shown in Figure 6b,d.

### 2.4. Cytocompatibility and Hemocompatibility of Hydrogels

**Figure 7:**
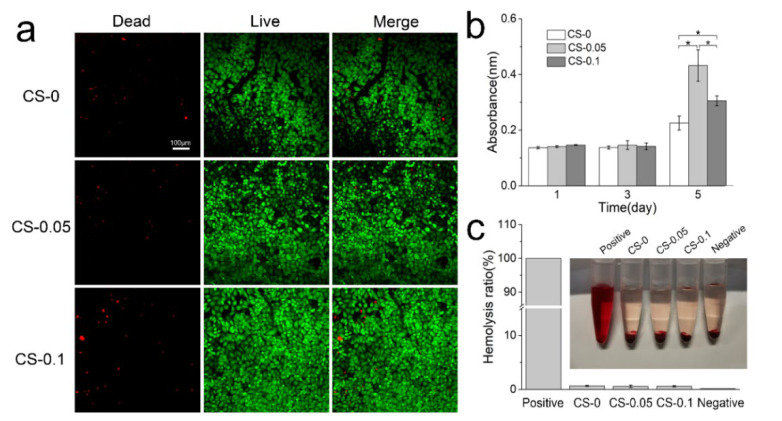
(**a**) Live and dead staining of HUVECs; (**b**) proliferation rates of HUVECs on different hydrogels; and (**c**) the hemolysis ratios of various hydrogels, * *p* < 0.05.

Cytocompatibility and hemocompatibility are essential criteria of hydrogels for biomedical applications. Herein, the cytocompatibility of hydrogels was evaluated via live and dead cell staining and CCK-8 proliferation assays. First, HUVECs were cultured on the surface of hydrogels for 4 days, then stained with a live and dead activity kit. Figure 7 shows the staining images under a confocal microscope. Cells in the CS-0.05 and CS-0.1 groups can adhere to the hydrogel surface and present a spindle-like morphology, which is consistent with that of the CS-0 group. Though few dead cells (labeled by the red color) can be found, the surviving rates in all groups exceed 95%. Notably, the coverage area of cells in the other two groups is larger than that of the CS-0 group, suggesting that CS addition can enhance the proliferation of HUVECs. To validate this conclusion, cell numbers from each group were measured after 1, 3 and 5 days via the CCK-8 kit, and the results are presented in Figure 7b. CS addition dramatically promotes the proliferation rate of HUVECs, leading to higher absorbance values than in the CS-0 group after 5 days. However, CS-0.05 presents the highest absorbance value, which may be ascribed to the competition among excess cells.

The hemocompatibility of hydrogels is identified in Figure 7c. Erythrocytes swell and burst in the positive group (DI water), while hydrogels and the negative group (PBS) show a similar canary yellow color to the normal saline. Quantitative evaluation reveals low hemolysis rates in all groups, which are below the internationally permitted level of 5% [24], indicating that these hydrogels do not cause serious hemolysis, and would be suitable for biomedical applications [25].

### 2.5. Modulation on Angiogenesis Behaviors

**Figure 8:**
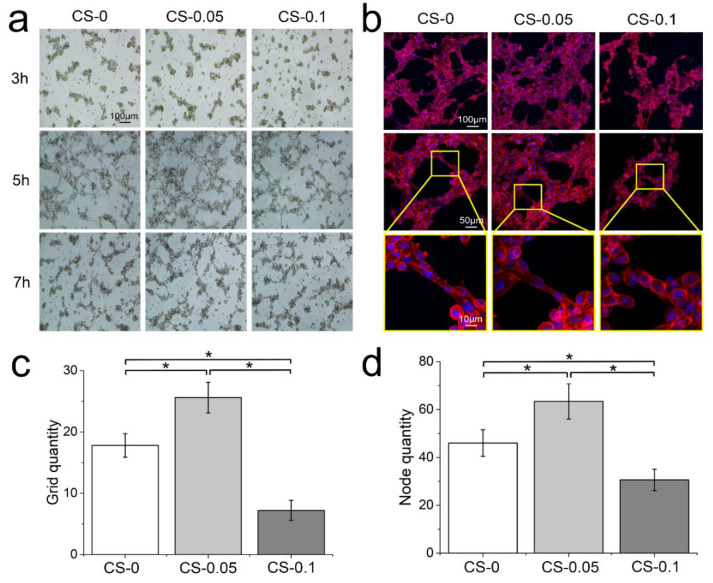
(**a**) Cell migration after 3 h, 5 h and 7 h of culture in sprouting assays; (**b**) fluorescent staining of nuclei and cytoskeletons after 5 h of culture; the numbers of (**c**) grids and (**d**) nodes in the tubular structure, * *p* < 0.05.

As a component of ECM, CS has been recognized to play multiple roles in regulating numerous cell activities [26]. To study the angiogenesis regulation of hydrogels on HUVECs, cells were photographed at different time points to observe dynamic changes during the sprouting assays. From Figure 8a, HUVECs are loose, without connections at the beginning of culture. After 5 h of culture, cells began to form networks with an elongated morphology, and the CS-0.05 group presents the most numerous and most dense networks. After 7 h, the tubular structures in all groups start to collapse and disappear. We further stained the cytoskeleton and nucleus at the 5 h time point, and observed cell morphology under laser confocal microscopy. Denser tubular structures and more elongated actin filaments were found in CS-0.05 group than in the other two groups. Meanwhile, in the CS-0.1 group, the angiogenesis ability of HUVECs was inhibited, with spherical and unstretched morphology at the junctions. The numbers of grids and nodes in the tubular structures shown in Figure 8b were counted via Image J software [27], and the results are presented in Figure 8c,d. As can be seen, the CS-0.05 group has the largest numbers of grids and nodes, and the CS-0.1 hydrogel has fewer grids and nodes than the CS-0 group, suggesting that a low concentration of CS might be more suitable for promoting angiogenesis and subsequent tissue regeneration.

CS has been demonstrated to participate in blood vessel formation [28]. Similar to other growth factors, CS suffers from rapid dissolution and biodegradation in human fluid, which greatly restricts its targeted effect. Instead of electrostatic absorption [19], we encapsulated CS in a genipin-crosslinked gelatin hydrogel to prolong its local release time. Additionally, the release profile of CS was tuned via different loading concentrations, without any obvious influence on its physical properties. It can be concluded that CS addition endows hydrogels with angiogenesis modulation through CS release according to the biological assays. However, excessive CS will occupy a higher proportion of the cell membrane, hindering the combination of other growth factors [29,30]. Thus, the results indicate that the CS-0.05 hydrogel possesses the highest proliferation number and the best angiogenesis behaviors of ECs among these groups, showing great potential for treating ischemic diseases and achieving tissue regeneration in terms of regulating angiogenesis behaviors.

It is noteworthy that multiple growth factors (VEGF, FGF-2, and TGF-β) are necessary for steps in angiogenesis [1,17], which leads to the collapsed angiogenesis structure after 7 h, even though the CS-0.1 hydrogel is able to release CS for more than 3 days. Therefore, the synergistic effect between different growth factors and cells should be further investigated to reveal the mechanism involved in vascular reconstruction.

## 3. Conclusions

Herein, a kind of functional hydrogel with CS release properties was proposed and tuned using various CS amounts to modulate the angiogenesis behavior of ECs. The obtained hydrogels showed excellent mechanical stability, cytocompatibility, and hemocompatibility. Among them, CS-0.05 hydrogel was able to dramatically enhance the angiogenesis behavior, with denser tubular structures and longer stability, which has potential in treating ischemic tissue diseases and accelerating tissue regeneration. Notably, this research also indicated that an excess amount of CS could weaken the angiogenesis process due to excessive binding on the cell membrane. Further research should take the synergistic effect between different growth factors and cells into consideration to reveal the underlying mechanism of angiogenesis.

## 4. Materials and Methods

### 4.1. Materials

Gelatin, genipin, and Azure A were purchased from Aladdin Biochemical Technology Co, Ltd. (Shanghai, China). Chondroitin sulfate (CS) was obtained from Aoxing Biology (Zhejiang, China). Human umbilical vein endothelial cells (HUVECs) were obtained from Beijing Oligobio (Beijing, China). Matrigel, RPMI 1640 medium, phosphate-buffered saline (PBS), and trypsin EDTA were products of Corning Incorporated Life Science (New York, NY, USA). Triton X-100, bovine serum albumin, and glycine were products of Solarbio Life Science (Beijing, China). CF568 phalloidin and DAPI were from Biotium (Fremont, CA, USA). The CCK-8 assay kit was bought from Dojindo (Kyushu, Japan), and the live/dead viability kit was a product of Nanjing Keygen Biotech. Co, Ltd. (Nanjing, China). Deionized (DI) water was obtained from a Mill-Q water purification system (Woburn, MA, USA) and was used in all experiments [23].

### 4.2. Fabrication of Hydrogels

An aqueous solution method was used to produce hydrogels. Briefly, gelatin was firstly dissolved in DI water at a concentration of 10 (*w*/*v*) %. Then, genipin (0.5 *w*/*w*% to gelatin) was added to the gelatin solution with gentle stirring. Three groups of CS (0, 0.05, and 0.1 *w*/*w*% to gelatin) were dissolved in DI water, separately. Subsequently, gelatin/genipin and CS solutions with different concentrations were mixed at 45 °C to form homogeneous systems and transferred to a Teflon mold at room temperature for 24 h. The obtained hydrogels were named CS-0, CS-0.05, and CS-0.1, respectively.

A TA rheometer (DHR2 Discovery, TA Instrument, Newcastle, USA) was employed to obtain the gelation time of different hydrogels. Then, 800 μL of hydrogel precursor solution was placed between the parallel plate with a gap of 1 mm. After 5 min, the time sweep tests were then performed at 25 °C with a constant frequency of 10 rad/s and strain of 1%.

### 4.3. Morphology Observation

The microstructure of lyophilized hydrogels was observed using a scanning electron microscope (FEI Quanta FEG 250, Hillsboro, OR, USA) with an accelerating voltage of 20 kV. Before observing, samples were fractured in liquid nitrogen and the cross-section of samples was sputtered with gold for 60 s to increase the conductivity during imaging.

### 4.4. Swelling Ratio and Biodegradation Properties

Swelling ratios of hydrogels were measured by immersing samples in PBS solution (pH = 7.4) at 37 °C. At pre-determined time intervals, samples were removed from the solution, dried superficially with filter paper, and weighed. The swelling ratio (SR) was determined using the following equation:

(1) $$\mathrm{Swelling}\;\mathrm{ratio}\;\left(\%\right)=\left[\left(W_{t}-{\;W}_{i}\right)/W_{i}\right]\;\times \;100\%$$

where $W_{i}$ is the initial weight of sample and $W_{t}$ is the weight of hydrogel after soaking. All tests were performed in triplicate.

Likewise, the degradation properties of hydrogels were determined by immersing samples in the PBS solution containing 7.5 unit/mL collagenase. The degradation rate (DR) was calculated through equation as follows:

(2) $$\mathrm{Degradation}\;\mathrm{rate}\;(\%)=\left[\left(W_{0}-{\;W}_{p}\right)/W_{0}\right]\;\times \;100\%$$

where $W_{0}$ is the initial mass of the sample and $W_{p}$ is the residues after degradation for pre-determined intervals.

### 4.5. X-ray Photoelectron Spectroscopy (XPS) Analysis

The chemical compositions of hydrogels were investigated by XPS (AXIS Supra, Japan) equipment. Elements were identified in the range of 0–1200 eV with a resolution of 1 eV. The atomic concentration of each element was calculated by determining the relevant integral peak intensities.

### 4.6. CS Release Behavior

Azure A dye was employed to determine the release profile of CS from hydrogels [22]. Briefly, 5 g Azure A powder was dissolved in 100 mL PBS to make a stock solution. The hydrogel extract was prepared by soaking 0.6 g sample in 2 mL PBS solution. At pre-determined time intervals, 100 μL extract solution from each group was mixed with 0.5 mL stock solution, and 100 μL fresh PBS was added back. The mixture was measured at a wavelength of 540 nm via a microplate reader (Nano-Drop 2000, Thermo Scientific, Waltham, MA, USA). Serially diluted stock solutions were also measured to obtain the standard curve.

### 4.7. Mechanical Test

The rheological test was employed to obtain the storage modulus (G’) of hydrogels under a dynamic frequency mode. Hydrogel samples were prepared as a disc 40 mm in diameter and 1 mm in height. Samples were investigated with a strain of 1% and the frequency ranged from 0.1 to 100 rad/s.

A universal testing machine (UTM2230, SUNS Technology, Shenzhen, China) equipped with a 100 N stress sensor was used for compressive tests at room temperature. A cylindrical hydrogel specimen with a diameter of 5 mm and a height of 10 mm was placed on the fixed plate. Crosshead was set at a speed of 5 mm/min. With the same procedure, data of ten cycles were recorded in the cyclic compressive tests. At least five samples were tested in each group.

### 4.8. Cytocompatibility and Hemocompatibility Assays

Before cell culturing, hydrogels from different groups were tailored into a circular shape and sterilized by soaking in ethanol overnight, followed by ultraviolet light irradiation [31]. Circular samples with the same weight were plated in 24-well plates, and HUVECs with a density of 50,000 cells/cm^2^ were seeded. The wells were then incubated at 37 °C in a humidified, 5% CO_2_ atmosphere with the culture medium being changed every other day. After 5 days of culture, a live/dead activity kit was then applied to evaluate the cytocompatibility as described in our previous study [32].

A cell counting kit-8 (CCK-8) was employed to investigate cell proliferation behavior on different hydrogels. The CCK-8 solution (10 *v*/*v*%) was added to each well after 1, 3, and 5 days of culture, respectively. After incubating for 2 h at 5% CO_2_ and 37 °C, 100 μL reaction mixture was transferred to a multi-well microplate reader and measured at the wavenumber of 450 nm.

A hemolysis test was performed in vitro using rabbit erythrocyte suspension according to the reported protocol [33]. Erythrocyte stock solution was made by diluting erythrocytes with PBS to 2 *v*/*v*%. Hydrogel extract (500 μL) from different hydrogels was added to the stock solution (500 μL) and incubated at 37 °C for 3 h. PBS and DI water were added to the stock solution and set as negative and positive controls, respectively. After incubation, the suspensions were centrifuged at 1000× *g* rpm for 10 min, and 200 μL supernatant was collected and determined at 540 nm by UV-vis spectrophotometer. The hemolysis rate was calculated as follows:

(3) $$\mathrm{Hemolysis}\;\mathrm{ratio}\;\left(\%\right)=\left[\left(A_{h}-{\;A}_{p}\right)/\left(A_{w}-{\;W}_{p}\right)\right]\;\times \;100\%$$

where $A_{h}$, $A_{w}$, and $A_{p}$ are the absorbance of samples reacted with leaching medium, PBS, and DI water, respectively. The protocol of animal experiments was approved by the Animal Ethics Committee of Zhengzhou University (No. SCXK 2021-0009).

### 4.9. In Vitro Sprouting Analysis

In vitro sprouting analysis was conducted according to the reported protocol [34]. Matrigel matrix was thawed at 4 °C and added to a confocal dish (200 μL per dish) on ice. The dishes were then incubated at 37 °C for 30 min to allow the Matrigel to gel. Cells with a density of 2 × 10^5^ cells/well were seeded. Then hydrogel extract (1 mL) from each hydrogel was added to wells. During the culture, cell morphology was captured under optical microscopy after 3, 5 and 7 h. After 5 h of culture, the cells were fixed with 4% paraformaldehyde and stained by CF568 phalloidin and DAPI and observed via laser confocal microscopy (LSM 880, Zeiss, Oberkochen, Germany). Angiogenesis behavior was evaluated by counting the number of grids and nodes in different fields via NIH ImageJ software [35]. At least 20 different areas were selected from each group.

### 4.10. Statistical Analysis

One-way analysis of variance (ANOVA) was performed to determine statistical significance. A *p*-value < 0.05 (denoted by an asterisk) was considered statistically significant.

## Data Availability

Not applicable.
